# Diffusion Tensors of Arbitrary-Shaped Nanoparticles in Fluid by Molecular Dynamics Simulation

**DOI:** 10.1038/s41598-019-55042-9

**Published:** 2019-12-12

**Authors:** Zi-Tong Zhang, Xin Zhao, Bing-Yang Cao

**Affiliations:** 1 Key Laboratory for Thermal Science and Power Engineering of Ministry of Education, Department of Engineering Mechanics, Tsinghua University, Beijing, 100084 China; 2 Beijing Key Laboratory of Space Thermal Control Technology, Beijing Institute of Spacecraft System Engineering, Beijing, 100094 China

**Keywords:** Characterization and analytical techniques, Nanoparticles

## Abstract

The anisotropic diffusive behavior of nanoparticles with complex shapes attracts great interest due to its potential applications in many fields ranging from bionics to aeronautic industry. Although molecular dynamics (MD) simulations are used widely to investigate nanoparticle diffusion properties, universal methods to describe the diffusion process comprehensively are still lacking. Here, we address this problem by introducing diffusion tensor as it can describe translational and rotational diffusion in three dimensions both individually and their coupling. We take carbon triple sphere suspended in argon fluid as our model system. The consistency of our results and velocity autocorrelation function(VAF) method validates our simulations. The coupling between translational and rotational diffusion is observed directly from analyzing diffusion tensor, and quantified by coupling diffusion coefficient. Our simulation reveals non-trivial effect of some factors in diffusion at nanoscale, which was not considered in previous theories. In addition to introducing an effective method to calculate the diffusion tensor in MD simulations, our work also provides insights for understanding the diffusion process of arbitrary-shaped particles in nanoengineering.

## Introduction

Nano-materials like graphene and carbon nanotubes have excellent properties, such as high thermal or electrical conductivity, good flexibility and large yield strength^1–3^. They are used widely as suspending nanoparticles in liquid medium for applications ranging from bionics, to medicine, to microelectronics, and to aeronautic industry, in which the motions of nanoparticles, including diffusion, orientational alignment and aggregation, play a significant role. For example, active particles that can autonomously propel due to their structures, thus can be used as motors to deliver drugs, antibody or other tiny stuffs by diffusing in living organism^4^. Nanoparticle suspensions can be used as thermal-responsive smart materials, as they can adapt their directional arrangements to the applied external field so as to display a tunable thermal conductivity that can be used for thermal control system for aerospace related applications^5^. Janus particles could assemble into hierarchical structures to due to their anisotropic properties that produces directional interactions with fluid and with each other^6^. The nanoparticles in external field also show attractive orientation behaviors^7,8^. Therefore, in order to develop better nanoparticle manipulation techniques, it is highly desirable to fully understand how the nanoparticles move and behave in fluid.

It is known that the behavior of particles in fluids can be treated as Brownian motion, a random motion of particles due to the collision by the fluid molecules. Einstein pointed out that nature of Brownian motion is diffusion of particles, including translational and rotational diffusion^9^. While the translational diffusion has been studied systematically in the past years^10,11^, the rotational diffusion still needs more attention since it is directly related to the particles’ orientational motion. For an isotropic particle such as a spherical particle, its diffusive behavior can be fully described by two parameters, translational and rotational diffusion coefficient. The rotational coefficient could be obtained from the classical Debye-Stokes-Einstein (DSE) relationship, ${D}_{r}=\frac{{k}_{b}T}{8\pi {a}^{3}\mu }$. Here *k*_*b*_ is the Boltzmann constant, *T* is temperature, *μ* is viscosity of surrounding fluid and *a* is the radius of spherical particle. The diffusion is much more complicated for anisotropic particles with complex shapes, as the translational diffusion and rotational diffusion are frequently coupled together. Brenner has pointed out that in this case the diffusion tensor should be used instead of a single diffusion coefficient^12,13^.

In the past years, most researchers used theoretical hydrodynamic method based on the continuum medium model to study the diffusion problem. A few models for particles of simple geometries, such as sphere, ellipsoid^14^ and rod^15^, produce accurate analytic solutions and has been confirmed by experimental data at macroscale. However, for cases such as anisotropic particles that are asymmetric in shape or when particles are subjected to external field or confinement, it becomes difficult to obtain exact solution by solving the equations. For the former, Garica *et al*. used many small beads to fill in the space that was occupied by the particle, then solved the equation sets of all the beads as the replacement^16^. They also developed a software Hydro++ to automatically compute dynamic properties of particles in fluid based on their ‘bead model’^17,18^. However, since the theory has large background for big spherical particles in a infinitely dilute fluid, it fails for nanoparticles, as found inconsistent with experiments^19–21^.

Numerous experimental work have been conducted to investigate the particle diffusive motion process and to check the validity of the classical theories for nanoparticles. In particular, high-speed particle motion tracking techniques was developed to determine the translational and rotational diffusion coefficient from the particle trajectories^22–25^. Deviation of experimental observation from classical theories even for simple-shaped particles were found in some cases^21,26^. Han *et al*., found the coupling between translational and rotational diffusion of ellipsoid was inconsistent with theoretical predictions^27^. On the other hand, many experiments require confinement in two-dimensional or quati-two-dimensional geometry as it is difficult to track the position and orientation of particles in three-dimensional space^22,23,28^, therefore can’t faithfully take into account the parameters concerned by industry, including size, aspect ratio and polarity of particles due to the restriction of experimental conditions.

With recent advances in computer technology, it is now possible to use molecular dynamics (MD) simulation method to provide detailed information on dynamics behavior of nanoparticles and fluid structures, with convenient tuning of particle geometrical parameters. Previous simulations show that nanoarticles diffusion significantly differs from macroparticles^29,30^. For the spherical particles, the effect of mass, size, and their interactions with fluid molecules have been discussed extensively^31–33^. The effects of specific environments (e.g. solid-liquid interface, liquid-liquid interface), states and surface properties of particles on the rotational diffusion have been extensively discussed^34,35^. At solid-liquid interface, particle diffusion is restricted and thus tend to rotate parallel to the solid^36^. Jose found the ratio of rotational correlation time to viscosity becomes much bigger than the hydrodynamic value near the isotropic-nematic transition for thermotropic liquid crystals^29^. Anisotropic diffusion can also be induced by the wettability asymmetry of the surfaces of particles as the nonwetting end rotates around the wetting ends^37^. In summary, results were found inconsistent with theoretical predictions quantitatively in many cases^26,30,38,39^.

However, these previous simulation work mainly focus on simple shaped particles, such as sphere, ellipsoid, rod and so on, while in reality nanoparticles are more complex shaped without good symmetry. Besides, as the single diffusion coefficient was used in simulation, inevitably it leads to loss of information on coupling between translational and rotational diffusion as well as its anisotropy in different directions, which is important in applications. To improve, researchers have alternatively used equivalent diffusion coefficients in specific directions (e.g. in-plane rotational diffusion coefficient)^39,40^, which benefits in obtaining general diffusion speed conveniently but with anisotropy neglected. Other methods via defining specific parameters such as rotation angle of rod at the major axis along displacement vector^37,41^, to which a cross comparison with others becomes difficult due to its customization. On the other hand, the diffusion tensor proposed by Brenner provides comprehensive information of diffusion process as we mentioned above, which is applicable to all types of arbitrary-shaped particles in fluid, and has been successfully applied in experimental studies of unsymmetrical particle diffusion, such as trimers and Boomerangs^22,24^.

Therefore, in this work, we introduce the diffusion tensor into MD simulations as an analysis method for diffusive motion of nanoparticle in fluid. Fick’s Law is used to calculate diffusion tensor of particle, from the relationship between diffusion tensor and displacement correlation function. Our simulation system is the nanoparticle composed of carbon triple spheres suspended in argon fluid. The nanoparticle is treated as a rigid body in order to compare with classical theoretical predictions. In the simulation, we systematically studied the effect of geometric parameters of particle including its size and angle between centers of each sphere. The coupling between translational and rotational diffusion was directly observed and further quantified from calculation of coupling diffusion coefficient. Moreover, the fact that our results slightly derivates from classical theories, indicating the subtle yet critical role of some microscopic factors that were often neglected in those previous theory but well captured by our simulation, such as slip boundary and molecular interactions. In addition to development of an effective method to calculate the diffusion tensor in MD simulations, our work also provides a basic understanding of nanoparticle diffusion.

## Computational Method

### Diffusion tensor

For rigid, spherical particle diffusing in fluid, its translational diffusion is quantified by translational diffusion coefficient^9^

1 $${D}_{t}=\frac{1}{6}\frac{\langle \Delta r{(t)}^{2}\rangle }{\Delta t},$$

here $\langle \ldots \rangle$ means ensemble average and Δ*r*(*t*) is the displacement of the center-of-mass position of the particles at time *t*. If the particle is asymmetrically-shaped, its diffusive motion is anisotropic and affected by its continuous change in orientation. A more detailed description on diffusion motion is essential.

Consider a system composed of N-nanoparticles with arbitrary shapes in fluid. Each particle’s motion has six independent parameters, so it requires six independent generalized coordinates, $\overrightarrow{q}=({q}_{1},{q}_{2},\ldots ,{q}_{6})$ to describe its dynamics behavior in six-dimensional configuration space. Generally $({q}_{1},{q}_{2},{q}_{3})$ are used to describe the position of the particle in physical space, and $({q}_{4},{q}_{5},{q}_{6})$ are used to describe the orientation of the particle. In this work, $({q}_{1},{q}_{2},{q}_{3})$ are chosen as the Cartesian coordinates of its center-of-mass position in the fixed lab coordinate frame. $({q}_{4},{q}_{5},{q}_{6})$ depend on the change of orientation, $d\overrightarrow{\varphi }$, which is defined in the particle own coordinate frame as the following way: the magnitude of it is the angle from its initial to final orientation and the direction of it is along the instantaneous axis of it. $d\overrightarrow{\varphi }$ is given as

2 $$d\overrightarrow{\varphi }={q}_{4}{\overrightarrow{e}}_{x}+{q}_{5}{\overrightarrow{e}}_{y}+{q}_{6}{\overrightarrow{e}}_{z}.$$

In the six-dimensional configuration space, the generalization of Fick’s law of diffusion is given as

3 $$\frac{\partial C}{\partial t}={D}_{ij}\frac{{\partial }^{2}C}{\partial {q}_{i}{q}_{j}}.$$

here, *C* is the concentration of particles in configuration space. The diffusion tensor *D*_*ij*_ is a second-rank tensor. It is more reasonable to treat the diffusive motion as a coupled translational and rotational diffusion instead of a single phenomenon in configuration space because of the physical nature as above stated.

Thus, ***D*** can be partitioned into four 3 × 3 sub-matrices as follows:

4 $${\boldsymbol{D}}=(\begin{array}{cc}{D}_{t} & {D}_{c}^{T}\\ {D}_{c} & {D}_{r}\end{array}).$$

here, *D*_*t*_ denotes the translational diffusion, *D*_*r*_ denotes the rotational diffusion, *D*_*c*_ denotes coupling between translation and rotation. The diffusion tensor is symmetrical and positive definite.

### Calculation of diffusion tensor

In N-nanoparticles systems, *q*_*i*_ will change during quite small-time interval under irregular forces from fluid atoms. Assuming that these particles don’t interact with each other and there are no external fields, the probability of change of *q*_*i*_ between Δ*q*_*i*_ and $\Delta {q}_{i}+d\Delta {q}_{i}$ is

5 $$W(\Delta {q}_{1},\Delta {q}_{2},\cdots \Delta {q}_{6};\Delta t)d\Delta {q}_{1}\cdots d\Delta {q}_{6}.$$

here *W* is probability density. It is symmetrical and normalized as follows:

6 $$W(\Delta {q}_{1},\Delta {q}_{2},\cdots \Delta {q}_{6};\Delta t)=W(\pm \Delta {q}_{1},\pm \Delta {q}_{2},\cdots \pm \Delta {q}_{6};\Delta t),$$

7 $$\int \cdots \int W(\Delta {q}_{1},\Delta {q}_{2},\cdots \Delta {q}_{6},\Delta t)d\Delta {q}_{1}\cdots d\Delta {q}_{6}=1.$$

According to continuity and conservation laws, the evolution of concentration *C* over a small time Δ*t* is given as

8 $$\begin{array}{c}C({q}_{1},\cdots {q}_{6},t+\Delta t)\\ =\int \cdots \int C({q}_{1}+\Delta {q}_{1},\cdots ,{q}_{6}+\Delta {q}_{6};t)W(\Delta {q}_{1},\cdots ,\Delta {q}_{6};\Delta t)d\Delta {q}_{1}\cdots d\Delta {q}_{6}.\end{array}$$

Expanding the left-hand side in Taylor series for small Δ*t*, and the right-hand side for small Δ*q*_*i*_, the equation is given as

9 $$\begin{array}{c}C+\frac{\partial C}{\partial t}\varDelta t+O{(\Delta t)}^{2}=\\ C\int \cdots \int Wd\Delta {q}_{1}\cdots d\Delta {q}_{6}+\frac{\partial C}{\partial {q}_{i}}\langle \Delta {q}_{i}\rangle +\frac{1}{2}\frac{{\partial }^{2}C}{\partial {q}_{i}\partial {q}_{j}}\langle \Delta {q}_{i}\varDelta {q}_{j}\rangle +O{(\Delta q)}^{3}.\end{array}$$

here, $\langle \ldots \rangle$ mean average changes for functions. Due to the symmetry of *W*, the mean displacement for each component of Δ*q* is

10 $$\langle \Delta {q}_{i}\rangle =0.(i=1,2\ldots 6)$$

$O{(\Delta t)}^{2}$ and $O{(\Delta q)}^{3}$ can be ignored, and we can get

11 $$\frac{\partial C}{\partial t}=\frac{1}{2}\frac{{\partial }^{2}C}{\partial {q}_{i}\partial {q}_{j}}\langle \Delta {q}_{i}\Delta {q}_{j}\rangle .$$

Comparing it to Eq.  3, we can obtain the relationship between coefficients of diffusion tensor and displacement correlation function, for each element of diffusion tensor **D**, the equation is

12 $${D}_{ij}=\frac{1}{2}\frac{\langle \Delta {q}_{i}\Delta {q}_{j}\rangle }{\Delta t},$$

where the summation convention is unused. In MD simulations, the ensemble average could be replaced by time average. $\Delta {q}_{i}(i=1,2,3)$ could be chosen as the center-of-mass displacement, and $\Delta {q}_{i}(i=4,5,6)$ could be defined as the following way to make calculation convenient. Introduce three mutually perpendicular unit vectors fixed on the particle ${u}_{k}(k=1,2,3)$, Δ*q*_*i*_ is given as

13 $$\Delta {q}_{i}=\frac{1}{2}\mathop{\sum }\limits_{k=1}^{3}{\overrightarrow{u}}_{k}(0)\times {\overrightarrow{u}}_{k}(t)(i=4,5,6).$$

This approximate method is accurate in short time^24,42^. The reason is as follows. The vector ${\overrightarrow{u}}_{k}(0)$ becomes ${\overrightarrow{u}}_{k}(t)$ at time *t*, and the relation between ${\overrightarrow{u}}_{k}(0)$ and ${\overrightarrow{u}}_{k}(t)$ is

14 $${\overrightarrow{u}}_{k}(t)={\bf{R}}(t){\overrightarrow{u}}_{k}(0).$$

here ${\bf{R}}(t)$ is the orthogonal rotation matrix at time *t*. Its components can be expressed with ${\overrightarrow{u}}_{k}$ as ${R}_{{k}_{1}{k}_{2}}={\overrightarrow{u}}_{{k}_{1}}(0)\cdot {\overrightarrow{u}}_{{k}_{2}}(t)({k}_{1},{k}_{2}=1,2,3)$, the cosine between the orientation vectors in time 0 and time *t*. It can be decomposed into two parts, the symmetric part ${{\bf{R}}}^{s}$, and the antisymmetric part ${{\bf{R}}}^{a}$. The equation is

15 $${\bf{R}}={{\bf{R}}}^{s}+{{\bf{R}}}^{a}.$$

where

16 $$\begin{array}{c}{{\bf{R}}}^{a}=\frac{{\bf{R}}-{{\bf{R}}}^{T}}{2}\\ =(\begin{array}{ccc}0 & {{\bf{R}}}_{12}^{a} & {{\bf{R}}}_{13}^{a}\\ -{{\bf{R}}}_{12}^{a} & 0 & {{\bf{R}}}_{23}^{a}\\ -{{\bf{R}}}_{13}^{a} & -{{\bf{R}}}_{23}^{a} & 0\end{array})\end{array}$$

${{\bf{R}}}^{a}$ has three indepent elements so it could be rewritten as a vector $\Delta \overrightarrow{u}$ whose components are as follows:

17 $$\Delta {u}_{p}(t)=-\,\frac{1}{2}{\varepsilon }_{pij}{{R}_{ij}}^{a}(t),p=1,2,3.$$

Here, ${\varepsilon }_{pij}$ is Levi-Civita symbol. So $$\Delta {u}_{p}(t)=[\begin{array}{ccc}{R}_{23}^{a} & -{R}_{13}^{a} & {R}_{12}^{a}\end{array}]$$. According to the definition of ${{\bf{R}}}^{a}$,

18 $$\Delta \overrightarrow{u}(t)=\frac{1}{2}\mathop{\sum }\limits_{k=1}^{3}{\overrightarrow{u}}_{k}(0)\times {\overrightarrow{u}}_{k}(t).$$

When time t is small enough, the magnitude of $\Delta \overrightarrow{u}$ is the angle from its original orientation to its final orientation. The direction of it is along the instantaneous axis of rotation consistent with our demand^43^. So we can take its components as $\Delta {q}_{i}(i=4,5,6).$

### Simulation details

The diffusive motion of nanoparticle in fluid is studied via molecular dynamics simulation. MD simulations could provide more detailed information including fluid-particle interaction and fluid structure than traditional theoretical method. We choose particles composed of three same carbon spheres suspended in argon fluid as simulation systems. For a single sphere, the Carbon atoms are uniformly arranged on the spherical surface. The distance between the carbon atom and the carbon atom is about 0.14 nm. The three spheres are arranged in contact with each other, and the whole particle is regarded as a rigid body. The shape of this particle could be determined by two parameters, the angle *θ* between centers of each sphere and the radius *r* of each sphere. *θ* and *r* could be adjusted over the simulation. The simulation system is confined in a 10.6 × 10.6 × 10.6 nm^3^ cubic box with three orthogonal Cartesian coordinates-oxyz. The boundary conditions in all three directions are periodic. The density *ρ*of argon are 1763 kg/m^3^ and temperature *T* is set as 300 K.

**Figure 1:**
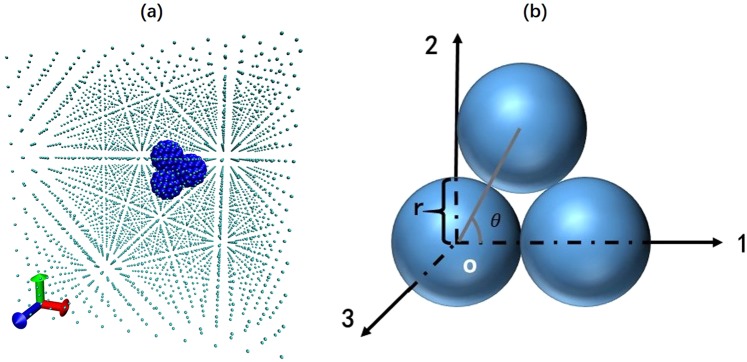
(**a**) Schematic of the triple carbon spheres in argon fluid. The cyan atoms are argon and the blue atoms are carbon. The particle is located in the center of this simulation system initially. (**b**) The reference frame of particle. *r* is the radius of each sphere, *θ* is the angle between centers of sphere.

Figure 1 shows the schematic representation of the triple carbon spheres in argon (Ar) fluid initially. The Ar atoms are uniformly distributed throughout the simulation system, and the nanoparticle is located in the center of system. The velocity and angular velocity of the nanoparticle are both set as zero and the velocity of Ar atoms is correspondent to Gaussian distribution at 300 K.

The Lennard-Jones (LJ) potential is selected to describe interaction between Ar and C atoms. LJ potential is simple in mathematical form and accurate enough for noble gas, applied well in MD simulations. The form of it is

19 $$\varphi (r)=4\varepsilon [{(\frac{\sigma }{r})}^{12}-{(\frac{\sigma }{r})}^{6}].$$

Here *ε* is potential wall and σ is collision radius, depending on the atom type. In this simulation, ${\varepsilon }_{Ar-Ar}=1.655\times {10}^{-21}\,{\rm{J}},\,{\sigma }_{Ar-Ar}=0.341\,{\rm{n}}{\rm{m}},\,{\varepsilon }_{C-Ar}=1.965\times {10}^{-21}\,{\rm{J}},\,{\sigma }_{C-Ar}=0.357\,{\rm{n}}{\rm{m}}.$^44^ *r* is the distance between the two interacting atoms. The nanoparticle is set as a rigid body to make comparison with theoretical predictions and to reduce the time-consuming of calculation^45^. This assumption has been widely used in theoretical analysis and simulations. The cut-off radius *r*_*c*_ is 2.5 nm. The NVT ensemble is used during the simulation. The temperature is controlled by Nose-Hoover thermostat. The time step in simulation is set as 1 fs. We run for 1000000 step and take the later 800000 step to do statistics. The simulation is performed by Lammps software^46^.

## Results and Discussion

**Figure 2:**
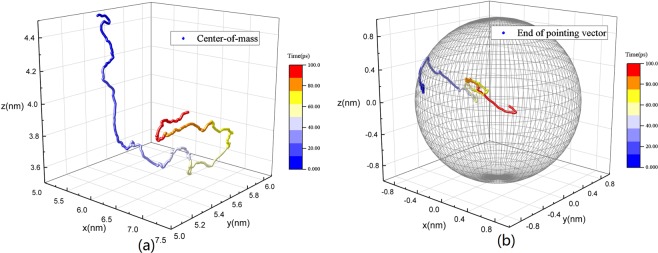
(**a**) The trajectories of center-of-mass of the particle in three dimensional space with color-coded in time. The unit vector $\,\overrightarrow{{e}_{1}}=(1,0,0)$ at time *t* = 0 is chosen as a pointing vector. (**b**) The trajectories of the end of the pointing vector moving on the surface of unit sphere with color-coded in time, which can indicate the changing of particle orientation.

The trajectories of the particle in three dimensions are shown in Fig. 2. From the overall movement of a particle, we can see the characteristics of typical Brownian motion: irregular and incessant translation in three dimensional physical space and rotation in orientational space under the random force from fluid molecules. The translational motion in all directions is unlimited while anisotropy exists, which is more obvious in rotation. In fact, the rotational motion is localized mainly in 1–2 plane during the time we observed. These phenomena are more clearly in the following discussion when we focus on the statistic mechanical characteristics, i.e. the mean-squared displacement of particle varied with time.

**Figure 3:**
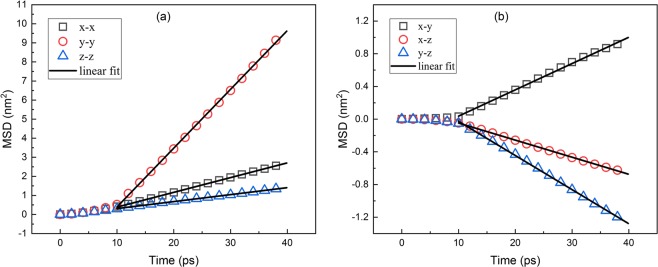
Translational Diffusion: Relationship between the mean-squared displacement (MSD) of the particle and time.

**Figure 4:**
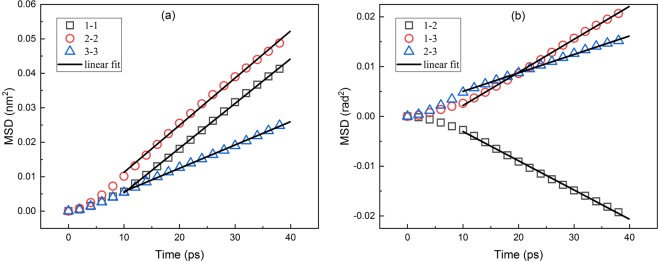
Rotational Diffusion: Relationship between the mean-squared angular displacement of the particle and time.

**Figure 5:**
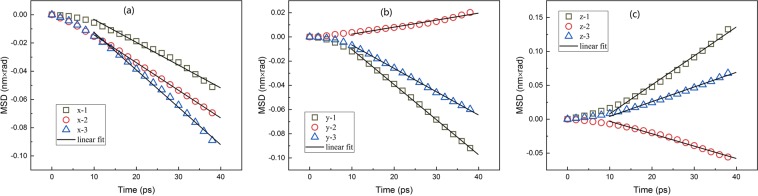
Coupling between translational and rotational diffusion: Relationship between the coupling displacement correlation functions of the particle and time.

Figure 3 shows the relationship between the mean-squared displacement (MSD) of the particle and time. At the initial stage, the MSD of the particle is parabolic with time and does not appear as a normal diffusion behavior. This is because of the imbalance forces from surrounding fluid atoms. And the asymmetrical shape and directionality of the particle at its original position may increase the effect. This process is called the ballistic regime^40,41^. After that the motion transform from ballistic to diffusive regime, MSD is proportional to time consistent with diffusion theory so the diagonal element of translational diffusion tensor can be calculated from the slope of fit. In addition, the translational diffusion in different directions are coupled together. The typical rotational diffusion behavior is also observed in Fig. 4. The shape of curves in Fig. 4 is similar to Fig. 3, which means the MSD in rotation is also proportional to time, similar to the translational part, consistent with classical theory. That proves that our definition of displacement in orientational space is valid in picosecond time scale of this simulation. In addition, the rotational diffusion differs more in different directions, especially in comparison of in-plane to out-plane, the difference mainly is due to its mass distribution. As shown in the trajectories, the mass of particle is mainly concentrated in 1–2 plane so that the in-plane rotational diffusion coefficient is small. The in plane coupling is negative while the coupling between in-plane and normal direction is positive. The diagonal and off- diagonal elements of rotational diffusion tensor can also be calculated from the slope of fit. In Fig. 5, we can observe the coupling between translational and rotational diffusion directly, which indicates the drag force along the particle will also lead to the rotation of the body. In previous works, the crossover from the anisotropic diffusion at short time to the isotropic diffusion at long time has been observed and Vasanthi *et al*. inferred that it is the coupling between translational and rotational diffusion that leads to this transformation^23,47^. Vivek *et al*. studied coupling and decoupling between translation and rotation of two different types of dimers by comparing the ratios of translational to rotational diffusion coefficients^48^. Coupling can be measured from calculating the coupling diffusion coefficients from the fit to the slope in Fig. 5 even the curves are not as ideal, i.e. pure translational or rotational diffusion.

To further validate our calculation, we compare the single translational diffusion coefficient *D*_*t*_ calculated by our method with the results calculated from velocity autocorrelation function (VAF). VAF is a classical method to determine particle diffusion coefficient, widely used in MD simulations.

20 $${D}_{t}=\frac{1}{3}{\int }_{0}^{\infty }\langle \overrightarrow{v}(t)\cdot \overrightarrow{v}(0)\rangle dt$$

**Figure 6:**
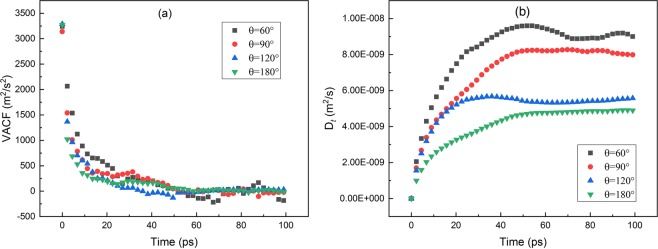
(**a**) Velocity autocorrelation function (VAF) varying with time; (**b**) The integral of VAF for calculating D_*t*_.

In Eq. (19) $\overrightarrow{v}$ means velocity of center-of-mass of the particle and *D*_*t*_ could be expressed as it. Figure 6(a) shows the VAFs changing as function of time, which converges after 60 ps then fluctuates around zero. In Fig. 6(b) the integrals of VAFs are calculated. The integral, which is *D*_*t*_ according to Eq. (19), increases then fluctuate around the certain value. The results are shown inTable 1. The maximum relative difference is within 16%. The consistency of our results for translational diffusion from the MSD and VAF methods indicates the good accuracy of our simulations.

**Table 1:** Translatioanl diffusion tensor for the cases with *R* = 0.7 nm.

*θ*(°)	D_*t_*MSD_(×10^−9^m^2^/s)	D_*t_*VAF_ (×10^−9^m^2^/s)	Relative Difference(%)
60	10.50	9.10	13.3
90	7.00	8.12	16.0
120	5.48	5.56	1.38
180	4.85	4.90	1.13

For the selected cases *r* = 0.7 nm and *θ* = 60°, the diffusion tensor is



Due to the asymmetry of the particle geometry, the values of the matrix diagonal elements are different. In-plane rotational diffusion is dominant. The diagonal elements of the diffusion tensor are all positive while the off-diagonal elements could be positive or negative. The coupling sub-matrix is not a zero-matrix. These coupling terms may erase the gap in diffusion of different directions, as the experimental work indicate the diffusion eventually become isotropic at long time. The size of the coupling terms is close to the uncoupling terms within an order of magnitude. In next part we will focus on the rotational diffusion.

We change the size of the sphere while maintaining *θ* = 60°, the rotational diffusion matrix is shown in Table 2. The rotational diffusion coefficients in all the three axis decrease monotonically as the particle diameter increases. That can be explained by DSE relationship qualitatively. It is easier for random forces from surrounding fluid atoms in different directions to counteract with each other simultaneously. These factors contribute to the reduction in diffusion coefficient. Particles of smaller radius are more sensitive to the change in size. With the increase of the diameter of particles, the dominant role of out-plane rotational diffusion further exemplify. The particle mass is mainly concentrated in 1–2 plane in all cases. The mass distribution becomes more unbalanced with larger radius of particle. Maybe the out-plane diffusion is less sensitive to collisions. Now, it is possible to produce nanoparticles with desirable sizes in industry. It is still need to tradeoff between the controllability in the external fields and diffusion efficiency of the particles. The results can serve as good references. The coupling terms have different characteristics. The value of these could be positive or negative without significant relation to size.

**Table 2:** Rotational diffusion tensor for the cases with *θ* = 60° and varying *R*.

*R*(nm)	D_11_(rad^2^/s)	D_22_(rad^2^/s)	D_33_(rad^2^/s)	D_12_(rad^2^/s)	D_13_(rad^2^/s)	D_23_(rad^2^/s)
0.7	1.20E + 09	1.37E + 09	6.76E + 08	−5.87E + 08	6.64E + 08	3.71E + 08
1	4.49E + 08	1.51E + 08	5.46E + 08	4.70E + 07	−8.92E + 07	−2.61E + 08
1.5	4.10E + 07	1.59E + 08	8.14E + 07	−8.70E + 05	5.14E + 07	−2.86E + 07
2	3.00E + 07	5.34E + 07	1.73E + 07	2.04E + 07	−4.10E + 07	−1.55E + 07
2.5	2.74E + 07	3.01E + 07	1.05E + 06	4.87E + 06	5.03E + 06	5.79E + 06

Then we change *θ* at *r* = 0.7 nm, the rotational diffusion matrix is shown in Table 3. As the angle between the centers of the spheres increases, the rotational diffusion coefficient along 1 axis increases while the rotational diffusion coefficient along 2 axis reduces gradually. This phenomenon can be explained qualitatively through the change of effective cross-sectional area. When *θ* increases, the section area of 1 axis decreases resulting in the decreases of effective radius, thus the rotation around 1 axis is easier and so as the diffusion coefficient along 1 axis increases. Meanwhile, the diffusion coefficient along 2 axis reduces as the section area of 2 axis increases. As the coupling between 1 and 2 axis exists, so strictly each part cannot be considered separately. It is notable that diffusion coefficient along 3 axis, which is out of the plane, reaches extreme value in the varying process. There is no dominating rotational direction. The influence of included angle is not as significant as the size of particle. In practical, diffusion properties can be adjusted by changing the block size, and modified by tuning the distribution, which is achievable nowadays in industry.

**Table 3:** Rotational diffusion tensor for the cases with *R* = 0.7 nm and varying *θ*.

*θ*(°)	D_11_(rad^2^/s)	D_22_(rad^2^/s)	D_33_(rad^2^/s)	D_12_(rad^2^/s)	D_13_(rad^2^/s)	D_23_(rad^2^/s)
60	1.20E + 09	1.37E + 09	6.76E + 08	−5.87E + 08	6.64E + 08	3.71E + 08
120	1.34E + 09	4.86E + 08	1.46E + 09	7.3E + 08	−2.4E + 08	−7.6E + 08
180	2.1E + 09	1.16E + 08	9.34E + 08	1.79E + 08	4.01E + 08	−3E + 08

Theoretical method based on continuous medium hypothesis, computes the diffusion tensor of particles in fluid by solving the Naiver-Stokes equation with stick boundary conditions. Garica *et al*. developed hydro++ software to calculate diffusion tensor in various environments which only requires to input solvent density, viscosity and shape of particles^18^. We use Hydro++ to calculate the diffusion tensor of particles which are under the same condition as the simulations. Change the size of the sphere when maintaining *θ* = 60°. The rotational diffusion matrix is shown in Table 4 and Change *θ* at *r* = 0.7 nm, the rotational diffusion matrix is shown in Table 5. Comparing the results from simulation and theoretical prediction, we find that they both monotonically decrease with the increase size of sphere. The predicted diffusion coefficients along x, y axes also are consistent with simulation results when theta changes. However, the theoretical results also have deviations from simulation. For example, theory often predict that rotation along different axes decouples, which contradicts to simulation results. The same problem has been observed in experiments. The values of diffusion coefficients from theoretical results are larger than simulation results, as previous studies show. That reminds us that diffusion behavior at nanoscale is different from macroscale and microscale. Some factors can be neglected in bulk but are shown to have non-trivial impact on diffusion of nanoparticles in fluid. For instance, fluid atoms can adsorb and form layered structures like solid at the surface of nanoparticles due to the molecular interaction between particle and fluid atoms. It increases the effective diameter of particle, therefore the rotational resistance which ultimately leads to the reduction of rotational diffusion coefficients. For macro-particles, these layers are relatively thinner as compared to particle diameter and the influence could be ignored. Secondly, the boundary condition is no longer strictly stick boundary when the size of particle is close to fluid molecules. Velocity slip occurs at particle surface but is not considered in Hydro++. In fact, the molecular interaction dominates over hydrodynamic interaction in nanoscale. DSE equation states that the diffusion coefficient depends only on the particle size *R*, viscosity *μ*, and temperature *T*. However at nanoscale, the diffusion coefficients of spherical particles made from different materials with nearly identical size exhibit huge difference. Traditional hydrodynamics method simplifies the interaction between atoms of particle and fluid into a single parameter, viscosity, which is appropriate in macroscale, but no so for nanoscale, as when the size of particles become close to fluid molecular, continuous medium hypothesis fails and this simplification no longer holds.

**Table 4:** Rotational diffusion tensor for the cases with *θ* = 60° and varying *R* by classical theory.

*R*(nm)	D_11_(rad^2^/s)	D_22_(rad^2^/s)	D_33_(rad^2^/s)	D_12_(rad^2^/s)	D_13_(rad^2^/s)	D_23_(rad^2^/s)
0.7	1.91E + 09	1.91E + 09	1.45E + 09	132.8	0	0
1	1.98E + 09	1.31E + 09	1.78E + 09	0.00E + 00	−3.90E + 08	0.00E + 00
1.5	5.67E + 08	4.30E + 08	5.67E + 08	0.00E + 00	3.65E + 00	0.00E + 00
2	2.39E + 08	1.82E + 08	2.39E + 08	0.00E + 00	8.24E + 00	0.00E + 00
2.5	1.22E + 08	9.29E + 07	1.22E + 08	0.00E + 00	−2.59E + 00	0.00E + 00

**Table 5:** Rotational diffusion tensor for the cases with $r=0.7\,{\rm{nm}}$ and varying $\theta$ by classical theory.

*θ*(°)	D_11_(rad^2^/s)	D_22_(rad^2^/s)	D_33_(rad^2^/s)	D_12_(rad^2^/s)	D_13_(rad^2^/s)	D_23_(rad^2^/s)
60	1.91E + 09	1.91E + 09	1.45E + 09	132.8	0	0
120	2.13E + 09	1.41E + 09	1.01E + 09	−6.2E + 08	0	0
180	3.07E + 09	8.64E + 08	8.64E + 08	0	0	0

## Conclusion

We introduce the diffusion tensor into MD simulations to study the rotational diffusive behavior of arbitrary-shaped nanoparticles suspended in fluid. The diffusion tensor is calculated from the relation between displacement correlation function and time, which is easy to trace and record in the MD simulations. The triple carbon spheres suspended in argon fluid is taken as our simulation system. The particle is treated as a rigid body to make a comparison with the traditional theoretical predictions. The consistency of our results for translational diffusion and VAF method indicates our simulations are valid. In our simulations, the coupling between translational and rotational diffusion is observed directly due to the asymmetric shape of the particle and the coupling diffusion coefficients in different directions could be calculated to qualify the coupling. The size of sphere and included angle between centers of spheres are changed during the simulations. The results provide good reference to adjust industrial particles for desired diffusive properties. The simulation results have nonnegligible deviation from the theoretical predictions of hydrodynamic methods. Some factors which are not considered in classical theories have non-trivial effect, e.g. the ordered structure of fluid atoms near the particle surface, the molecular interactions. The assumptions of hydrodynamic theories, the continuous medium hypothesis and stick boundary situations fail in nanoscale. So the viscosity in the DSE equation is not enough to describe the diffusive behavior. This work provides an effective method to determine diffusion properties of nanoparticle arbitrary-shaped via MD simulations. It could be applied to nanoengineering in terms of nanoparticle diffusion in further studies.

## Supplementary information

supplement materials

## Data Availability

All data presented in the during the current study are available from the corresponding author on reasonable request.
