# Supplementary material for: Diffusion Tensors of Arbitrary-Shaped Nanoparticles in Fluid by Molecular Dynamics Simulation
Source: Sci Rep. 2019 Dec 12;9:18943. doi: 10.1038/s41598-019-55042-9 (PMC6908649; doi:10.1038/s41598-019-55042-9)
Supplement: Supplementary file 1 — supplement materials [file 41598_2019_55042_MOESM1_ESM.pdf]

Diffusion Tensors of Arbitrary-Shaped Nanoparticles in Fluid by Molecular Dynamics Simulation

Zi-Tong Zhang (张梓彤)[1], Xin Zhao(赵欣)[2], Bing-Yang Cao (曹炳阳) [1,*]

[1]Key Laboratory for Thermal Science and Power Engineering of Ministry of Education, Department of Engineering Mechanics, Tsinghua University, Beijing 100084, China

[2]Beijing Key Laboratory of Space Thermal Control Technology, Beijing Institute of Spacecraft System Engineering, Beijing 100094, China

*Corresponding author: Tel/Fax: +86-10-6279-4531; E-mail: caoby@tsinghua.edu.cn

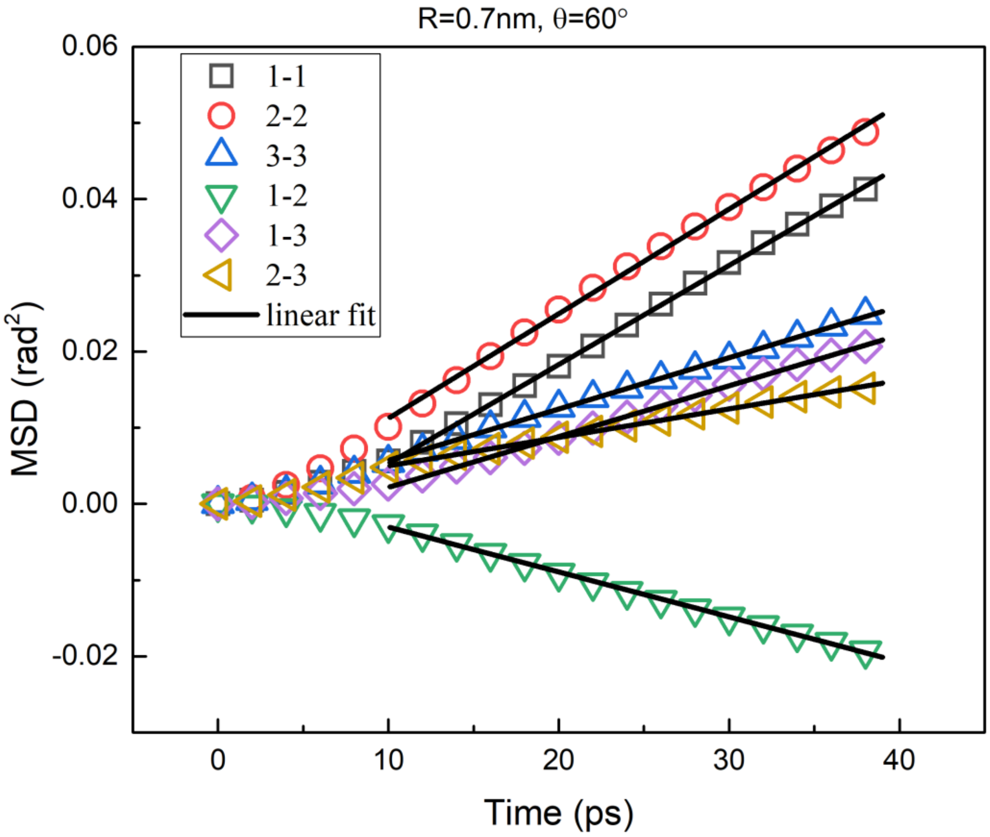

FIG. S1. Relationship between the mean-squared displacement and time for R=0.7 nm, $\theta = 60°$

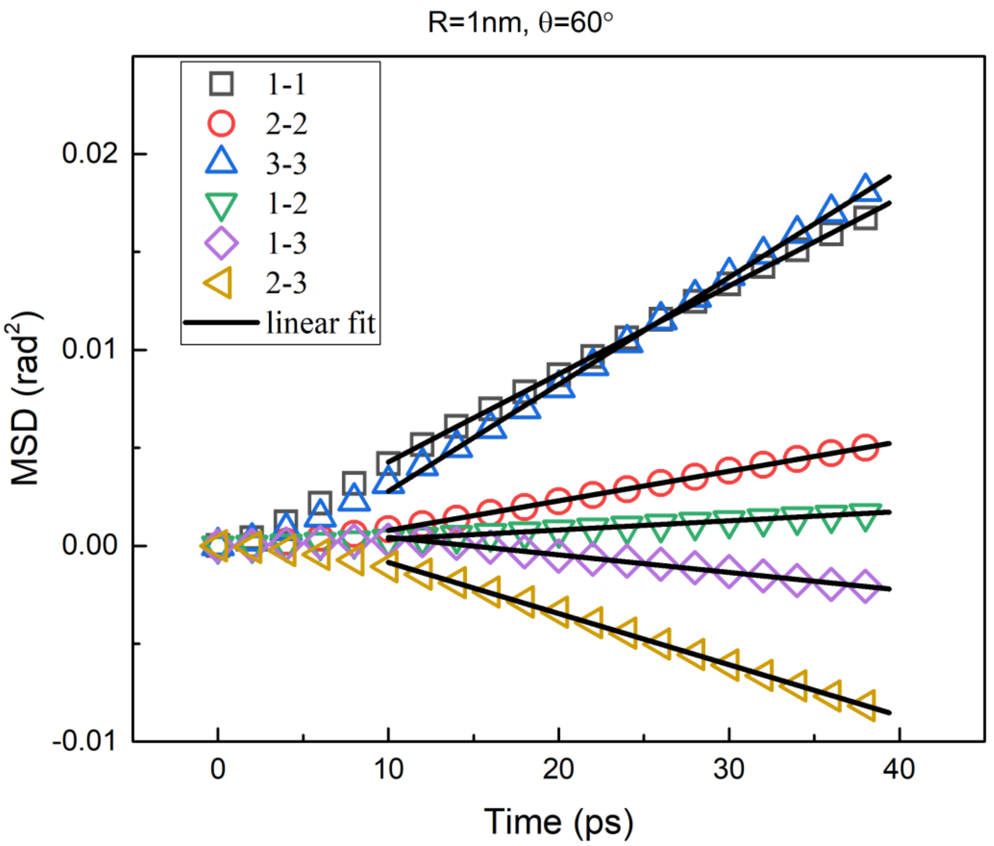

FIG. S2. Relationship between the mean-squared displacement and time for R=1 nm, $\theta = 60°$

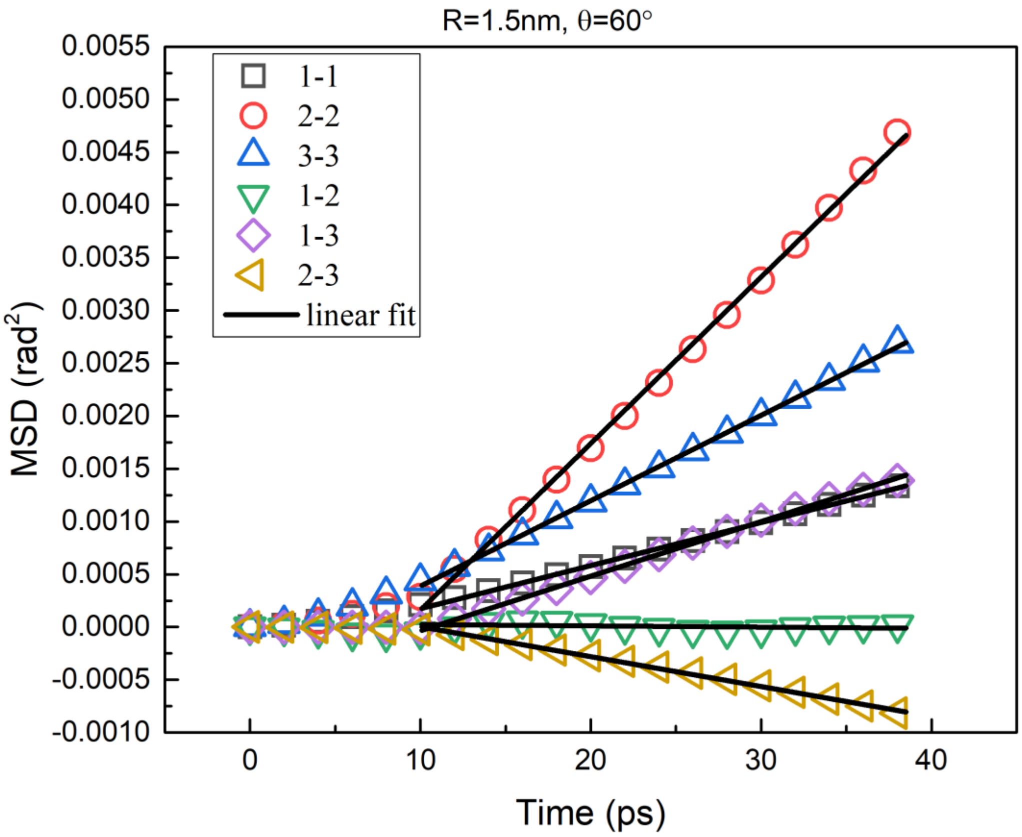

FIG. S3. Relationship between the mean-squared displacement and time for R=1.5 nm, $\theta = 60°$

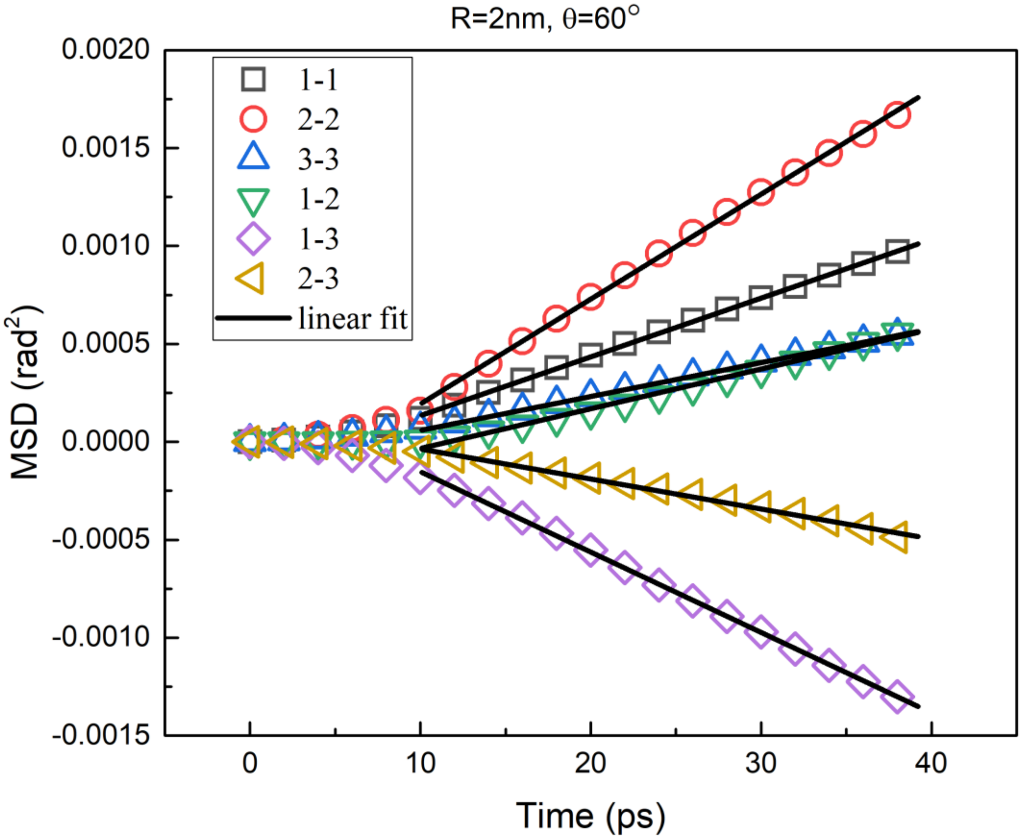

FIG. S4. Relationship between the mean-squared displacement and time for R=0.7 nm, $\theta = 60°$

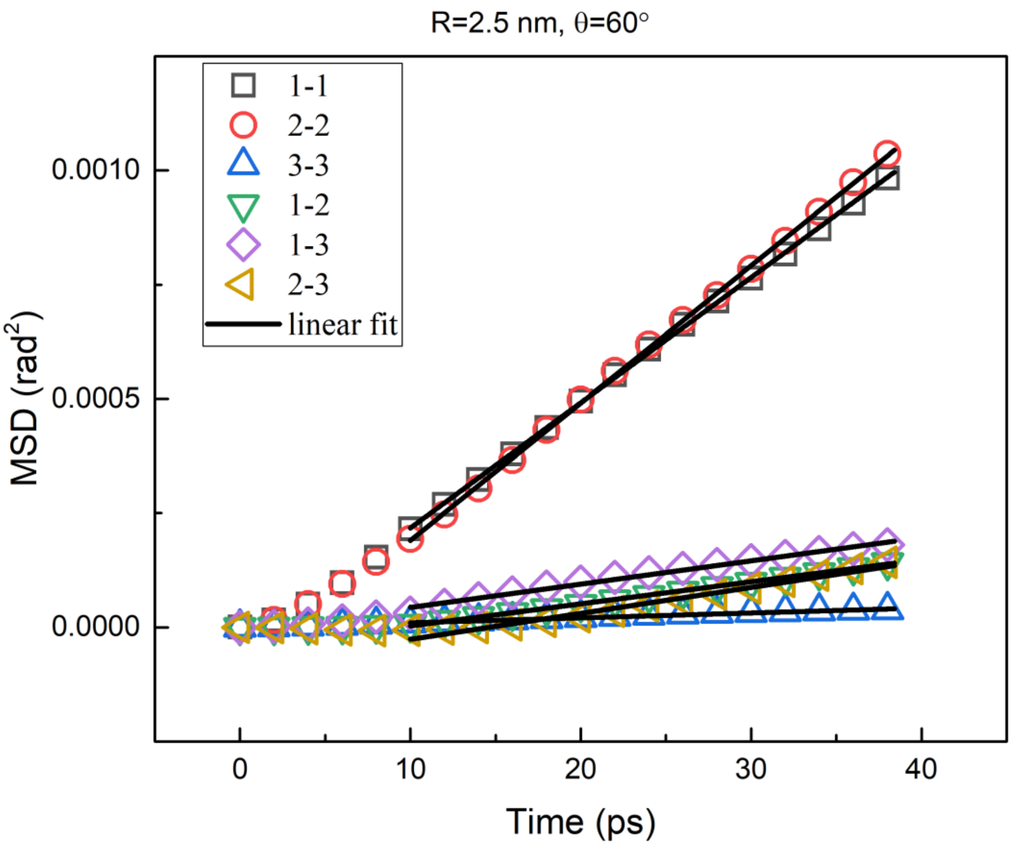

FIG. S5. Relationship between the mean-squared displacement and time for R=2.5 nm, $\theta = 60°$

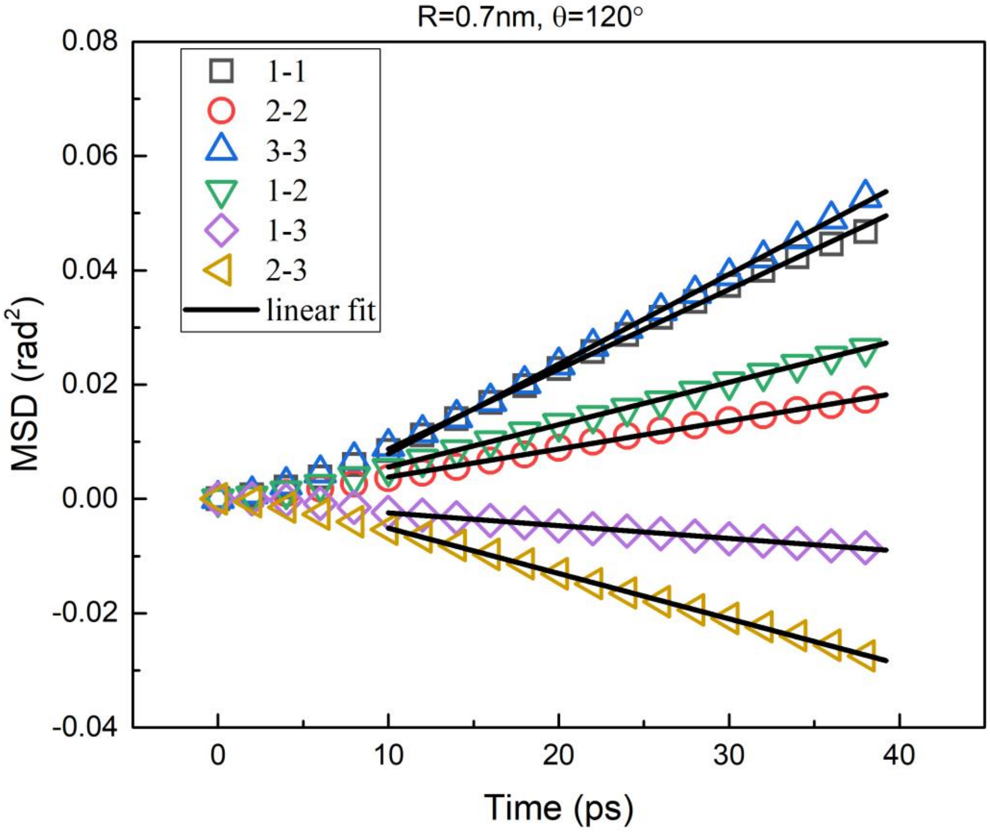

FIG. S6. Relationship between the mean-squared displacement and time for R=0.7 nm, $\theta = 120°$

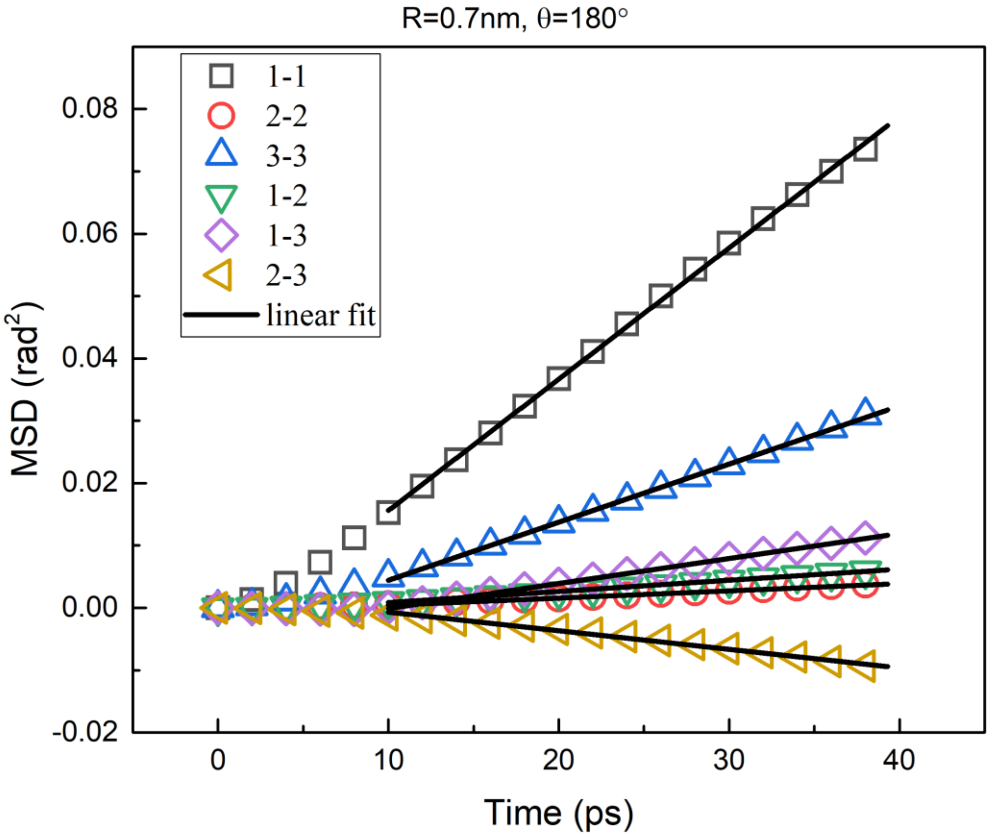

FIG. S17 Relationship between the mean-squared displacement and time for R=0.7 nm, $\theta = 180°$
